# Identification of paediatric cancer patients with poor quality of life

**DOI:** 10.1038/sj.bjc.6604826

**Published:** 2008-12-09

**Authors:** L Sung, R J Klaassen, D Dix, S Pritchard, R Yanofsky, B Dzolganovski, R Almeida, A Klassen

**Affiliations:** 1 Division of Haematology/Oncology, The Hospital for Sick Children, 555 University Avenue, Toronto, ON, M5G 1X8, Canada; 2 Department of Pediatrics, Children's Hospital of Eastern Ontario, 401 Smyth, Ottawa, ON, K1H 8L1, Canada; 3 Department of Pediatrics, University of British Columbia, A119D, 4480 Oak Street, Vancouver, BC, V6H 4C9, Canada; 4 Department of Pediatrics, University of British Columbia, A123D, 4480 Oak Street, Vancouver, BC, V6H 3V4, Canada; 5 Department of Pediatrics and Child Health, University of Manitoba, 675 McDermot Avenue, Winnipeg, MB, R3E 0V9, Canada; 6 Child Health Evaluation Sciences, The Hospital for Sick Children, 424, 123 Edward Street, Toronto, ON, M5G 1E2, Canada; 7 Department of Pediatrics, McMaster University, HSC 3N27, 1200 Main Street West, Hamilton, ON, L8S 4J9, Canada

**Keywords:** quality of life, children, chemotherapy

## Abstract

The primary objective was to describe predictors of physical, emotional and social quality of life (QoL) in children receiving active treatment for cancer. This Canadian multi-institutional cross-sectional study included children with cancer receiving any type of active treatment. The primary caregiver provided information on child physical, emotional and social QoL according to the PedsQL 4.0 Generic Core scales. Between November 2004 and February 2007, 376 families provided the data. In multiple regression, children with acute lymphoblastic leukemia had better physical health (OR: 0.37, 95% CI 0.23, 0.60; *P*<0.0001) while intensive chemotherapy treatment (OR: 2.34, 95% CI: 1.42, 3.85; *P*=0.0008) and having a sibling with a chronic condition (OR: 2.53, 95% CI: 1.54, 4.15; *P*=0.0002) were associated with poor physical QoL. Better emotional health was associated with good prognosis, less intensive chemotherapy treatment and greater household savings, whereas female children and those with a sibling with a chronic condition had poor social QoL. Physical, emotional and social QoL are influenced by demographic, diagnostic and treatment variables. Sibling and household characteristics are associated with QoL. This information will help to identify children at higher risk of poor QoL during treatment for cancer.

Measurement of quality of life (QoL) in paediatric cancer patients is becoming increasingly emphasized in clinical trials. This outcome is important as it provides a measure of well-being from the perspective of the parent or child. To date, most QoL research in paediatric cancer has been directed towards the development of reliable and valid instruments and describing expected QoL in this population. Ultimately, better understanding of QoL in children with cancer may be useful for several reasons. This knowledge may help parents and children anticipate the expected course of events during treatment. In addition, measurement of QoL may help families and healthcare professionals choose specific treatment strategies, if these strategies are associated with similar survival rates but different expected QoL. Another goal, though, may be to identify a group of children with expected poor QoL such that they could be targeted for supportive care interventions to improve their health.

There are many challenges to measuring QoL in children. First is the issue of self-report *vs* proxy report. Although self-report is the usual method for assessing QoL, this method is not always feasible or possible for children with health conditions who may be too ill, unwilling or lacking the necessary language skills, attention span or cognitive abilities to complete questionnaires themselves. Paediatric cancer patients are often very young, which emphasises difficulty with self-report. Proxies, usually the parent, may need to provide information on their behalf (Matza *et al*, 2004; Eiser and Jenney, 2007). Eiser and Morse (Eiser and Morse, 2001a, 2001b) have systematically reviewed parent proxy and child self-report of QoL and found that parents and children typically agreed upon more observable phenomenon, such as the level of physical activity, functioning and symptoms. Conversely, poor agreement was seen in more subjective areas, such as social or emotional functioning (Eiser and Morse, 2001a, 2001b). Although parent and child responses may differ, others have emphasised that parent perspectives are important and valid perspectives of the child's QoL (Matza *et al*, 2004; Pickard *et al*, 2004; Eiser and Jenney, 2007). As parents are often the primary reporters of symptoms and are the main decision-makers on behalf of their child, their evaluation of QoL is clinically relevant, when used in a complementary manner to child self-report when obtainable (Matza *et al*, 2004; Pickard *et al*, 2004; Eiser and Jenney, 2007).

Second, choosing a specific instrument to measure QoL in a study is challenging. In general, generic and disease-specific instruments are available (Guyatt *et al*, 1993). Disease-specific measures may be most useful when responsiveness and sensitivity are important. However, generic instruments also are important as there are normative reference data and their use permits comparison across different patient populations (Spieth and Harris, 1996). For our purpose, the PedsQL generic module represented a particularly attractive generic measure as it has been widely used in children with cancer and there are enough normative data and previous experience to allow us to delineate a group with particularly poor QoL.

Most of the literature attempting to predict QoL in children with cancer has focused on late effects of cancer. However, QoL during active treatment also is important to children and their families. Studies of children receiving active treatment primarily have consisted of small studies that did not identify those at high risk of poor outcomes (Nathan *et al*, 2004). Consequently, the primary objective of this report was to describe predictors of physical, emotional and social QoL in children receiving chemotherapy for cancer. The secondary objective was to identify children at risk for particularly poor QoL as defined as physical, emotional and social QoL that was at least two standard deviations below the mean for a general paediatric population.

## Materials and methods

### Patients

This study was a subset of a larger study designed to evaluate psychosocial health in parents of children receiving chemotherapy for cancer (Klassen *et al*, 2008). Children were eligible for inclusion if they were receiving treatment for any type of malignancy, if they were initially diagnosed more than 2 months before enrollment on this study, and if they were not considered palliative as defined as no reasonable chance for cure by their healthcare team. In addition, children were only eligible if the parent respondent was the person most responsible for the day-to-day decision-making for that child for the past year and the parent respondent could read English. Children were enrolled from five tertiary care Canadian paediatric cancer centres as follows: BC's Children's Hospital (Vancouver), CancerCare Manitoba (Winnipeg), Children's Hospital of Eastern Ontario (Ottawa), The Hospital for Sick Children (Toronto) and McMaster Children's Hospital (Hamilton).

### Methods

Patients were approached for participation either in the in-patient or outpatient settings in a consecutive fashion. The family was given a booklet to complete in which questions about the child's QoL were asked (see Outcomes below). The booklet also contained questions about child, parent and family characteristics. The completed booklet was returned to the team in person or by mail. Child variables included demographic information, and information on diagnosis, treatment and parent-reported prognosis (very good or excellent *vs* good, fair or poor). We asked parents about the intensity of treatment, which was scored on a 5-point rating scale (more intensive (4 or 5) *vs* less intensive (1–3)). Both the prognosis and intensity of treatment scales were developed for this study. We also asked whether the child had a chronic condition other than cancer. Parent variables included demographics, highest education of the primary caregiver and their spouse, employment and marital status and whether the primary caregiver had a chronic condition. Family variables included whether a sibling had a chronic condition, household income and savings.

Categorisation for chronic conditions in children and adults was derived from those used in the National Longitudinal Survey of Children and Youth (NLSCY; Statistics Canada, 2002). The NLSCY is a long-term study conducted by Statistics Canada, which follows the development and well-being of Canadian children from birth to early adulthood. Household income was recoded into above and below $60 000 annually (approximate median value in this sample). We also computed adjusted family income, a measure of income that adjusts for family size and composition that accounts for the benefits of multiple wage earners in the family as well as the economy of multiple individuals living in a single household compared with per capita income (Carson, 2002).

In addition to the data provided by parents, diagnosis and treatment information were abstracted from hospital charts by institutional clinical research associates. Institutional research ethics approval was obtained from each of the participating centers and written informed consent was obtained from all participants.

### Outcomes

The PedsQL is a multidimensional instrument that is reliable and valid in healthy populations and in children with cancer (Varni. *et al*, 1998a, 1998b, 1999a, 1999b, 2001, 2002). This instrument is composed of a 23-item PedsQL 4.0 Generic Core scale that reflects four dimensions, namely physical, emotional, social and school functionings. In general, the summary scores available from the PedsQL are psychosocial and physical scores, with psychosocial scores consisting of emotional, social and school dimensions. Because children on cancer chemotherapy often do not attend school, we could not assess the school domain for the majority of children, and consequently, we did not determine the psychosocial summary score. Thus, the outcomes for this study were the physical, emotional and social summary scores of the PedsQL 4.0 Generic Core Scales. A 1-month recall period was used.

We examined dimension scores in two ways. First, to determine what factors were associated with the three dimension scores, we examined the scores as continuous variables, which maximized the ability to identify predictors of these outcomes assuming that the relationship between the predictors and outcomes was linear. We presented these associations as *β*-coefficients with their standard errors as derived from linear regression. Positive *β*-coefficients meant that the predictor (or increasing values of the predictor) was associated with better QoL, whereas negative *β*-coefficients suggested that the predictor (or increasing values of the predictor) was associated with worse QoL. Second, we also wanted to determine predictors of clinically significant impairment of physical, emotional and social QoL. Varni *et al* (2003) previously explored the cutoff points for ‘at-risk status’ for impaired QoL and determined in a large paediatric population, that one standard deviation below the mean of the total population sample was a clinically meaningful measure of impaired QoL as it represented scores similar to children with severe chronic health condition. However, because our population consisted of children with a chronic health condition (namely cancer), we defined low QoL as a score of two standard deviations below the population mean;(Varni *et al*, 2003); these values were derived using data from a PedsQL database (Varni *et al*, 2007b).

To see whether the model could distinguish between children with and without poor physical, emotion and social QoL, we compared items from the PedsQL 3.0 Acute Cancer Module for these 2 groups and based on clinical experience, hypothesized that those who were at high risk for poor physical health should have worse pain and hurt scores, those at high risk for poor emotional health should have worse pain and hurt, nausea, anxiety, worry and cognition scores and those at high risk for poor social health should have worse scores on the communication and cognition domains.

### Statistics

Potential predictors of physical, emotional and social scores were determined using univariate linear or logistic regression analyses depending on whether the outcome was QoL as a continuous or dichotomous variable. For multiple regression modelling, factors that were associated with QoL at *P*<0.1 were entered into a forward selection model. All statistical analyses were performed using the SAS statistical program (SAS-PC, version 9.1; SAS Institute Inc., Cary, NC). All tests of significance were two-sided, and statistical significance was defined as *P*<0.05.

## Results

Subjects were enrolled between November 2004 and February 2007. A total of 513 parents were asked to participate in this study and 501 agreed. We received completed questionnaires back from 412 parents, giving an overall response rate of 80.3%. We excluded data for one parent who answered the questionnaire retrospectively, leaving a sample size of 411. Of these 411, 376 had children who were at least 2 years of age and could complete the PedsQL assessment. Parent and child characteristics appear in Table 1. Of the 376 children, 275 (73.1%) had leukemia or lymphoma and most children had acute lymphoblastic leukemia (ALL; 214/376, 56.9%). The remaining children had a solid tumour (55/376, 14.6%) or a brain tumour (35/376, 9.3%). Disseminated disease was present in 27/376 (7.2%) of the entire cohort and was present in 17/55 (30.9%) of those with solid tumours. The majority of parents rated their child's prognosis as very good or excellent (293/376; 77.9%). The most common chronic conditions reported for parents were anxiety (65/376; 17.3%), respiratory allergies (62/376; 16.5%) and depression (57/376; 15.2%). The most common chronic conditions reported for siblings were non-food allergies (35/376; 9.3%), mental handicap (13/376; 3.5%) and heart problems (10/376; 2.7%).

The mean summary score for physical function was 54.9 (95% CI: 9.4, 96.9; *N*=374), which was 28.4 points lower than the mean physical function score of 83.3 found in a large statewide pediatric survey in which QoL was assessed in 10 241 families (Varni *et al*, 2003). Similarly, the mean summary scores for emotional QoL was 61.1 (95% CI: 25, 95; *N*=376), which was 19.2 points lower than the mean emotional score of 80.3 in the statewide survey (Varni *et al*, 2003), and the mean summary scores for social QoL was 69.7 (95% CI: 35, 100; *N*=372), which was 12.5 points lower than the mean social score of 82.2 in the statewide survey (Varni *et al*, 2003). In our study, poor QoL was defined as QoL that was at least two standard deviations below the mean for a general paediatric population. Two standard deviations below the population mean varied by child age, but this value ranged from 36.82 to 61.59 for physical function, whereas the value ranged from 41.42 to 56.82 for emotional scores and 32.07 to 60.63 for social scores (Varni *et al*, 2007b). The number of children with particularly poor physical, emotion and social QoL as defined as scores at least two standard deviations below the population mean were 143/374 (38.2%), 94/376 (25%) and 51/372 (13.7%), respectively.

Table 2 illustrates that the child factors associated with better physical health were younger child age, ALL, better parent-rated prognosis, absence of radiotherapy or surgery to remove cancer, less intensive chemotherapy treatment and absence of other child chronic conditions. Parent factors that were associated with better child physical health were younger parent age, and the absence of a chronic condition in the parent. Finally, if the sibling had a chronic condition, then the child's physical health was worse. Tables 2 also illustrates the factors that were associated with emotional and social QoL in univariate linear regression.

In examining multiple regression models, only child age was included if both child age and parent age met criteria for inclusion into the model because they were highly correlated (Spearman *r*=0.61; *P*<0.0001). Similarly, if both ALL and any surgery to remove cancer met criteria for inclusion in the multiple regression model, only ALL was included because they were highly negatively correlated (Spearman *r*=−0.61; *P*<0.0001). Finally, in terms of family factors, if both adjusted income and savings of at least $10 000 both met criteria to be included in multiple regression, only adjusted income was used as they were highly correlated (Spearman *r*=0.52; *P*<0.0001).

Variables that were independently associated with physical, emotional and social QoL appear in Table 3. Older child age and more intensive chemotherapy treatment both were independently associated with worse physical, emotional and social functions. Interestingly, if the child's parents reported that the sibling or parent had a chronic condition, this occurrence was associated with worse function in the child with cancer; those with a parent with a chronic condition has worse physical and emotional functions, whereas those with a sibling with a chronic condition had worse physical and social functions.

Tables 4 and 5 illustrate logistic regression analyses to examine predictors of poor physical, emotional and social QoL as defined as having a score less than two standard deviations below the population mean. In multiple logistic regression analyses, a diagnosis of ALL independently predicted for better physical function, whereas those receiving intensive chemotherapy treatment as perceived by the parent, and those with a sibling with a chronic condition had a two- to three-fold increased odds for poor physical QoL (Table 5). In terms of emotional QoL, those with a parent-rated prognosis of very good or excellent and those with savings of at least $10 000 had better emotional function, whereas those receiving intensive chemotherapy treatment were at an almost five-fold increased odds of poor emotional function. Finally, female children and having a sibling with a chronic condition increased the odds of poor social function (Table 5).

Table 6 shows that groups predicted to be at high risk for poor physical, emotional and social QoL according to the multiple regression models had worse scores on the PedsQL acute cancer module domains hypothesized to be associated with physical, emotion and social health.

## Discussion

We determined variables independently associated with physical, emotional and social QoL in children receiving active treatment for cancer. In addition, we were able to delineate a group of children at particularly high risk of poor QoL as defined as two standard deviations below the mean score for healthy children. As expected, when QoL was examined as a continuous (rather than dichotomous) variable, regression analyses yielded a greater number of significant predictors of QoL. This information is helpful for gaining insight into what factors impact on QoL for children with cancer.

However, predicting low QoL probably is clinically more relevant to identify high-risk children who may be targeted for future supportive care interventions. We found that children with ALL had better physical function, whereas those whose parents reported more intensive chemotherapy treatment and those with siblings with a chronic condition had poorer physical QoL. Parent self-reported worse prognosis, more intensive chemotherapy treatment and smaller household savings all predicted worse emotional function. Finally, being female and having a sibling with a chronic condition predicted for poor social function.

Our finding that children with ALL had better physical function than children with other types of cancers is not surprising and is consistent with other reports (Meeske *et al*, 2004; Varni *et al*, 2007a). It also is clinically intuitive that more intensive chemotherapy treatment would be associated with worse physical and emotional functions.

We also found that chronic diseases in the sibling and parent were associated with the child's QoL. This influence of parent and sibling chronic condition on the child's QoL was consistent among the different analyses. More specifically, those with a sibling with a chronic condition had particularly poor physical and social QoL. Others have shown that parents with poor physical or psychosocial health have children with poorer QoL in several different conditions (Silver *et al*, 1998; Waters *et al*, 2000; Wagner *et al*, 2003; Williams *et al*, 2003). A study in paediatric cancer also found an association between maternal depression and child self-reported poor QoL (Vance *et al*, 2001). We were not able to identify literature that specifically examined sibling chronic conditions and paediatric cancer QoL, and thus our finding is unique.

Several possibilities may explain these associations. First, there may be a biological explanation; the chronic conditions may be heritable, and thus may be causative in the child with cancer's poor QoL. However, this hypothesis is not supported by our data as we did not find an association between the child with cancer having a chronic condition and worse QoL. Second, it is possible that parents who perceive their own health or the health of their other children as worse may also perceive that the child with cancer has worse QoL. Finally, it is possible that having a sibling with a chronic condition is somehow causative in poor physical and social QoL related to effects on the home environment.

A limitation of our study is that we used only parent-reported QoL. Parent and child report are known to differ, especially for the psychosocial domains of health (Eiser and Morse, 2001a). However, in our study, proxy report was necessary as a large proportion of the sample were too young to provide self-report data. In addition, others have noted that the multiple different perspectives of QoL including the parent perspective are all important and contribute to our understanding of child health (Pickard *et al*, 2004). Nonetheless, future research should focus on child self-report to understand whether such an approach results in fundamentally different high-risk groups compared with parent proxy respondents. Second, we modelled several different outcomes, and therefore there is a possibility of chance findings. These analyses were all conducted as secondary objectives, and therefore these results must be considered hypothesis generating. Third, we developed the prognosis and intensity of therapy scales for this study, and therefore there are no reported data on their reliability or validity. Finally, as with any study that attempts to build a prediction model, similar studies in different populations of children on active treatment for cancer will be critical to examine model validation and to determine the best variables to delineate a high-risk population at risk for poor QoL.

The results of this study are important as the majority of QoL research in paediatric cancer has focused on late effects of cancer therapy, or examined both children on and off treatment rather than during active therapy. However, QoL during active treatment also is important to study and will influence the experiences of the child and family. We would expect that QoL should be very different between children receiving active treatment *vs* those who have completed treatment, as the treatment period is predominated by toxicities of therapy and often radical changes in normal day-to-day routines for the child and family. Conversely, QoL in survivors would be expected to be mostly influenced by sequelae of therapy. Both of these perspectives are important and improvement in QoL during both periods should be a priority. Our report is valuable as it is the largest study to our knowledge focused on QoL in children receiving active treatment for cancer.

One of the difficulties with trying to measure QoL in children receiving active treatment for cancer is that symptoms during chemotherapy often fluctuate greatly depending on when QoL is assessed relative to when chemotherapy is administered, as well as the specific treatments provided. This issue is most relevant for aggressive chemotherapy that is typically administered in cycles every 2–4 weeks. Our use of a 1-month recall period was an attempt to try and capture in a summary form the effects of these different phases of symptoms. However, future research within specific diagnostic subgroups will be able to better define the contribution of specific chemotherapy protocols and agents to QoL.

The strengths of our study include that we sampled paediatric cancer patients from multiple centers, and thus have data that are generalisable. In addition, we have one of the largest cohorts in which sufficient clinical covariates were obtained such that we were able to built prediction models of the child's QoL. To the best of our knowledge, this is the first description of defining children at risk for particularly poor QoL.

This information may be used in different ways. First, we will be able to use the information on predictors to target specific groups for interventional supportive care trials. For example, we could target children receiving more aggressive chemotherapy treatments for interventions to improve their physical and emotional health. Second, these associations raise new hypotheses about contributors to the health of children with cancer. For example, the associations between chronic conditions in the parent and siblings raise the possibility that health of the family may affect the health of the child with cancer.

In summary, physical, emotional and social QoL are influenced by demographic, diagnosis, and treatment variables. In addition, characteristics of the sibling and household are associated with QoL. This information can be used to identify children at higher risk of poor QoL during active treatment for cancer.

## Figures and Tables

**Table 1:** Demographics of study population

**Variable**	**N^a^**	**Value**
*Child factors*		
Age, no. (%)	376	
2–4 years		110 (29.3)
5–7 years		87 (23.1)
8–12 years		84 (22.3)
⩾13 years		95 (25.3)
Median years since diagnosis (IQR)	376	0.6 (0.3, 1.3)
Male (%)	376	215 (57.2)
Cancer diagnosis, no. (%)	376	
Leukemia		233 (62.0)
Lymphoma		42 (11.2)
Neuroblastoma		19 (5.1)
Sarcoma		36 (9.6)
Brain		35 (9.3)
Other		11 (2.9)
No. acute lymphoblastic leukemia (%)	375	214 (57.1)
No. acute myeloid leukemia (%)	376	13 (3.5)
No. relapse (%)	376	31 (8.2)
No. prognosis of very good or excellent (%)	363	293 (80.7)
No. received radiation (%)	376	82 (21.8)
No. received any surgery to remove cancer (%)	376	79 (21.0)
No. received SCT (%)	376	30 (8.0)
No. intensive chemotherapy treatment (%)	370	110 (29.7)
No. received alternative treatment (%)	374	32 (8.6)
		
*Parent factors*		
Median parent age in years (range)	368	39.1 (20.2, 75.9)
No. male (%)	375	47 (12.5)
No. first parenting experience (%)	369	168 (45.5)
No. smoke (%)	376	67 (17.8)
No. university degree (%)	376	120 (31.9)
No. working (%)	371	163 (43.9)
No. chronic condition (%)	376	215 (57.2)
No. married (%)	376	311 (82.7)
		
*Household factors*		
No. sibling with chronic condition (%)	376	110 (29.3)
No. annual household income ⩾$60 000 (%)	354	182 (51.4)
Median adjusted household income in dollars (IQR)	349	28 846 (17 500, 42 500)
No. savings ⩾$10 000 (%)	330	164 (49.7)

IQR=interquartile range.

a The total sample size of 376; some respondents did not complete all questions.

**Table 2:** Univariate predictors of summary physical, emotional and social dimension scores^a^

	**Physical summary score**		**Emotional summary score**		**Social summary scores**	
**Variable**	***β*±s.e.^b^**	***P*-value**	***β*±s.e.^b^**	***P*-value**	***β*±s.e.^b^**	***P*-value**
*Child factors*						
Age in years	−1.74±0.26	<0.0001	−0.85±0.21	<0.0001	−0.91±0.22	<0.0001
Time since diagnosis	−0.70±0.98	0.48	0. 00±0.80	1.00	−2.53±0.83	0.003
Male	3.75±2.66	0.16	3.80±2.09	0.07	5.14±2.14	0.02
Acute lymphoblastic leukemia	13.28±2.57	<0.0001	5.83±2.08	0.005	5. 66±2.15	0.009
Relapse	−5.03±4.70	0.29	−1.73±3.78	0.65	−10.35±3.89	0.008
Prognosis of very good or excellent	10.73±3.36	0.002	10.73±2.63	<0.0001	10.10±2.70	0.0002
Radiation	−9.41±3.15	0.003	−4.02±2.51	0.11	−4.42±2.60	0.09
Surgery to remove cancer	−14.61±3.14	<0.0001	−3.84±2.55	0.13	−7.01±2.60	0.008
Stem cell transplant	−7.57±4.83	0.12	−0.83±3.84	0.83	−6.25±3.92	0.11
Intensive chemotherapy treatment	−15.06±2.79	<0.0001	−12.96±2.18	<0.0001	−7.65±2.32	0.001
Alternative treatment	−3.55±4.63	0.44	−7.90±3.72	0.03	−7.23±3.79	0.06
Other chronic condition	−9.46±3.44	0.006	−6.72±2.73	0.01	−3.27±2.81	0.25
						
*Parent factors*						
Age in years	−0.66±0.19	0.0006	−0.16±0.15	0.31	−0.19±0.16	0.23
Male	3.00±3.98	0.45	7.02±3.13	0.03	2.68±3.22	0.41
Smoke	−4.67±3.45	0.18	−4.20±2.71	0.12	−4.21±2.79	0.13
University Education	4.37±2.81	0.12	1.63±2.23	0.47	4.55±2.28	0.05
Working	4. 23±2.67	0.11	4. 32±2.10	0.04	5.63±2.15	0.009
Chronic condition	−10.14±2.61	0.0001	−9.13±2.05	<0.0001	−8.68±2.11	<0.0001
Married	6.02±3.46	0.08	5. 84±2.74	0.03	7.14±2.79	0.01
						
*Household factors*						
Sibling with chronic condition	−11.30±2.83	<0.0001	−4.19±2.28	0.07	−8.57±2.32	0.0002
Annual household income ⩾$60 000	2.18±2.69	0.42	−1.25±2.13	0.56	5.24±2.20	0.02
Adjusted household income	0.00±0.00	0.30	0.00±0.00	0.86	0.00±0.00	0.006
Savings ⩾$10 000	1.11±2.78	0.69	0.83±2.17	0.70	4.30±2.20	0.05

s.e.=standard error.

a Mean summary scores are presented in the Results.

b *β*-Coefficient from univariate linear regression. Positive *β*-coefficients meant that the predictor (or increasing values of the predictor) was associated with better quality of life, whereas negative *β*-coefficients suggested that the predictor (or increasing values of the predictor) was associated with worse quality of life.

**Table 3:** Multiple linear regression for physical, emotional and social summary scores^a^

**Variable**	***β*±s.e.^b^**	***P*-value**
*Physical summary score*		
Age of the child	−1.37±0.26	<0.0001
Acute lymphoblastic leukemia	5.93±2.50	0.02
Intensive chemotherapy treatment	−6.12±0.90	<0.0001
Parent with chronic condition	−5.45±2.40	0.02
Sibling with chronic condition	−8.52±2.56	0.001
		
*Emotional summary score*		
Age of the child	−0.68±0.19	0.0005
Male child	3.80±1.88	0.04
Prognosis of very good or excellent	7.84±2.38	0.001
Intensive chemotherapy treatment	−5.47±0.72	<0.0001
Male parent	6.17±2.82	0.03
Married	4.96±2.50	0.05
Parent with chronic condition	−5.29±1.94	0.007
		
*Social summary score*		
Age of the child	−0.94±0.22	<0.0001
Time since diagnosis	−3.52±0.94	0.0004
Male child	5.14±2.07	0.01
Intensive chemotherapy treatment	−3.93±0.81	<0.0001
Parent working	5.72±2.11	0.01
Sibling with chronic condition	−5.90±2.22	0.02
Household adjusted income	0.00±0.00	0.004

s.e.=standard error.

a Mean summary scores are presented in the Results.

b *β*-coefficient from univariate linear regression, Positive *β*-coefficients meant that the predictor (or increasing values of the predictor) was associated with better quality of life, whereas negative *β*-coefficients suggested that the predictor (or increasing values of the predictor) was associated with worse quality of life.

**Table 4:** Predictors of low physical, emotional and social summary scores^a^^,^^b^

	**Physical summary score**		**Emotional summary score**		**Social summary score**	
**Variable**	**OR (95% CI)**	***P*-value**	**OR (95% CI)**	***P*-value**	**OR (95% CI)**	***P*-value**
*Child factors*						
Age in years	1.07 (1.02, 1.12)	0.003	0.99 (0.95, 1.04)	0.80	0.99 (0.93, 1.05)	0.77
Time since diagnosis	0.98 (0.84, 1.15)	0.79	0.92 (0.75, 1.12)	0.39	1.16 (0.96, 1.41)	0.12
Male	0.70 (0.46, 1.06)	0.09	1.08 (0.67, 1.73)	0.76	0.44 (0.24, 0.80)	0.008
Acute lymphoblastic leukemia	0.35 (0.23, 0.53)	<0.0001	0.77 (0.48, 1.22)	0.26	0.99 (0.54, 1.79)	0.97
Relapse	1.12 (0.53, 2.34)	0.75	0.70 (0.28, 1.77)	0.45	2.06 (0.84, 5.09)	0.12
Prognosis of very good or excellent	0.48 (0.28, 0.82)	0.007	0.34 (0.20, 0.60)	0.0001	0.59 (0.29 1.19)	0.14
Radiation	1.44 (0.88, 2.37)	0.15	1.13 (0.65, 1.97)	0.67	0.76 (0.35, 1.62)	0.47
Any surgery to remove cancer	3.04 (1.83, 5.07)	<0.0001	0.78 (0.43, 1.42)	0.42	1.35 (0.68, 2.68)	0.39
Stem cell transplantation	2.26 (1.06, 4.81)	0.03	1.32 (0.58, 2.98)	0.51	1.29 (0.47, 3.53)	0.62
Intensive chemotherapy treatment	2.59 (1.64, 4.10)	<0.0001	4.09 (2.49, 6.72)	<0.0001	1.76 (0.95, 3.25)	0.07
Alternative treatment	1.20 (0.58, 2.48)	0.62	1.90 (0.89, 4.06)	0.10	1.92 (0.78, 4.71)	0.15
Other chronic condition	1.87 (1.09, 3.20)	0.02	1.18 (0.65, 2.16)	0.58	0.59 (0.24, 1.45)	0.25
						
*Parent factors*						
Age in years	1.02 (0.99, 1.06)	0.099	0.98 (0.95, 1.02)	0.28	0.97 (0.92, 1.01)	0.14
Male	0.73 (0.38, 1.39)	0.33	0.68 (0.31, 1.46)	0.32	0.39 (0.12, 1.31)	0.13
Smoke	1.44 (0.84, 2.46)	0.18	1.48 (0.83, 2.63)	0.19	0.99 (0.46, 2.15)	0.98
University Education	0.73 (0.47, 1.16)	0.18	0.77 (0.46, 1.28)	0.31	0.97 (0.51, 1.83)	0.92
Working	0.66 (0.43, 1.01)	0.06	0.70 (0.43, 1.14)	0.15	0.50 (0.26, 0.94)	0.03
Chronic condition	1.83 (1.19, 2.82)	0.008	1.97 (1.20, 3.24)	0.007	1.98 (1.04, 3.75)	0.04
Married	0.91 (0.53, 1.58)	0.75	0.59 (0.33, 1.05)	0.07	0.85 (0.40, 1.79)	0.67
						
*Household factors*						
Sibling with chronic condition	2.35 (1.49, 3.70)	0.0002	1.44 (0.88, 2.37)	0.15	1.88 (1.02, 3.46)	0.04
Annual household income ⩾$60 000	0.89 (0.58, 1.37)	0.59	0.70 (0.43, 1.15)	0.16	0.68 (0.37, 1.25)	0.21
Adjusted household income per $100 000	1.00 (0.88, 1.14)	0.97	0.90 (0.77, 1.04)	0.15	0.95 (0.79, 1.14)	0.57
Savings ⩾$10 000	0.98 (0.63, 1.53)	0.93	0.63 (0.38, 1.06)	0.08	0.78 (0.41, 1.46)	0.44

CI=confidence interval; OR=odds ratio.

a Mean summary scores are presented in the Results.

b Low is defined as ⩽2 s.d. below the mean for a general paediatric population (see Methods).

**Table 5:** Multiple logistic regression for low physical, emotional and social summary scores^a^^,^^b^

**Variable^c^**	**OR (95% CI)^a^**	***P*-value**
*Physical summary score*		
Diagnosis of not acute lymphoblastic leukemia	2.67 (1.67, 4.28)	<0.0001
Intensive chemotherapy treatment	2.34 (1.42, 3.85)	0.0008
Sibling with chronic condition	2.53 (1.54, 4.15)	0.0002
		
*Emotional summary score*		
Prognosis of not very good or excellent	2.66 (1.39, 5.11)	0.003
Intensive chemotherapy treatment	4.90 (2.78, 8.64)	<0.0001
Less than $10 000 of savings	1.77 (1.00, 3.12)	0.0496
		
*Social summary score*		
Child female	2.27 (1.22, 4.23)	0.01
Sibling with chronic condition	2.12 (1.13, 3.96)	0.02

OR=odds ratio; CI=confidence interval.

a Mean summary scores are presented in the Results.

b Low is defined as ⩽2 s.d. below the mean for a general paediatric population (see Methods).

c potential predictors shown in terms of factors associated with worse quality of life (e.g., in this table, female child gender is shown rather than male child gender).

**Table 6:** Comparison of PedsQL acute cancer module items hypothesised to be different between those predicted to be at high and low risk for poor physical, emotional and social functionings

**Variable**	**High risk group^a^** **Median (IQR)**	**Low risk group Median (IQR)**	***P*-value^b^**
*Physical summary score*	*N*=19	*N*=357	0.037
Pain and hurt	50.0 (37.5, 62.5)	62.5 (50.0, 75.0)	
			
*Emotional summary score*	*N*=8	*N*=368	
Pain and hurt	25.0 (18.8, 56.3)	62.5 (50.0, 75.0)	0.01
Nausea	25.0 (12.5, 37.5)	55.0 (40.0, 75.0)	0.0003
Anxiety	29.2 (12.5, 37.5)	50.0 ( 25.0, 75.0)	0.08
Worry	50.0 (29.2, 50.0)	75.0 (50.0, 100.0 )	0.01
Cognition	43.3 (17.5, 50.0)	68.8 (50.0, 87.5)	<0.0001
			
*Social summary score*	*N*=41	*N*=335	
Communication	50.0 (33.3, 75.0)	75.0 (50.0, 91.7)	0.0005
Cognition	55.0 (41.7, 65.0	70.0 (50.0, 87.5)	0.0002

IQR=interquartile range; PedsQL=paediatric quality of life inventory.

a Predicted high-risk group defined as those with attributes identified as independently associated with poor functioning in logistic multiple regression (from Table 5).

b *P*-value for comparison between high- and low-risk groups using Wilcoxon rank-sum test.
